# Chronic Stress Triggers Expression of Immediate Early Genes and Differentially Affects the Expression of AMPA and NMDA Subunits in Dorsal and Ventral Hippocampus of Rats

**DOI:** 10.3389/fnmol.2017.00244

**Published:** 2017-08-10

**Authors:** Anibal Pacheco, Felipe I. Aguayo, Esteban Aliaga, Mauricio Muñoz, Gonzalo García-Rojo, Felipe A. Olave, Nicolas A. Parra-Fiedler, Alexandra García-Pérez, Macarena Tejos-Bravo, Paulina S. Rojas, Claudio S. Parra, Jenny L. Fiedler

**Affiliations:** ^1^Laboratory of Neuroplasticity and Neurogenetics, Faculty of Chemical and Pharmaceutical Sciences, Department of Biochemistry and Molecular Biology, Universidad de Chile Independencia, Chile; ^2^Department of Kinesiology, Faculty of Health Sciences, Universidad Católica del Maule Talca, Chile; ^3^Faculty of Medicine, School of Pharmacy, Universidad Andres Bello Santiago, Chile

**Keywords:** stress, dorsal hippocampus, ventral hippocampus, NMDA receptor subunits, AMPA receptor subunits, immediately early genes, c-Fos, Arc

## Abstract

Previous studies in rats have demonstrated that chronic restraint stress triggers anhedonia, depressive-like behaviors, anxiety and a reduction in dendritic spine density in hippocampal neurons. In this study, we compared the effect of repeated stress on the expression of α-amino-3-hydroxy-5-methyl-4-isoxazolepropionic acid (AMPA) and N-methyl-D-aspartate (NMDA) receptor subunits in dorsal and ventral hippocampus (VH). Adult male Sprague-Dawley rats were randomly divided into control and stressed groups, and were daily restrained in their motion (2.5 h/day) during 14 days. We found that chronic stress promotes an increase in c-Fos mRNA levels in both hippocampal areas, although it was observed a reduction in the immunoreactivity at pyramidal cell layer. Furthermore, Arc mRNAs levels were increased in both dorsal and VH, accompanied by an increase in Arc immunoreactivity in dendritic hippocampal layers. Furthermore, stress triggered a reduction in PSD-95 and NR1 protein levels in whole extract of dorsal and VH. Moreover, a reduction in NR2A/NR2B ratio was observed only in dorsal pole. In synaptosomal fractions, we detected a rise in NR1 in dorsal hippocampus (DH). By indirect immunofluorescence we found that NR1 subunits rise, especially in neuropil areas of dorsal, but not VH. In relation to AMPA receptor (AMPAR) subunits, chronic stress did not trigger any change, either in dorsal or ventral hippocampal areas. These data suggest that DH is more sensitive than VH to chronic stress exposure, mainly altering the expression of NMDA receptor (NMDAR) subunits, and probably favors changes in the configuration of this receptor that may influence the function of this area.

## Introduction

The hippocampus is a heterogeneous structure along its dorso-ventral axis, displaying a differentiated synaptic network with other brain areas, which determines its influence in different functions (Fanselow and Dong, 2010; Strange et al., 2014). Lesion studies in rodents have indicated that dorsal hippocampus (DH; posterior in primates) is implicated in cognitive functions, such as learning, memory and spatial memory acquisition (locomotion, navigation and orientation; Moser et al., 1993; Fanselow and Dong, 2010). In contrast, lesion of ventral hippocampus (VH; anterior in primates) reduces both anxiety-like behaviors (Kjelstrup et al., 2002) and freezing responses (Richmond et al., 1999), revealing that VH has a role in emotional and motivated behaviors (Fanselow and Dong, 2010; Strange et al., 2014). Recent studies revealed electrophysiological differences between DH and VH, that probably determine their specific functions (Malik et al., 2016).

On the other hand, hippocampus has a lamellar structure along its entire dorso-ventral axis, characterized by a well-defined tri-synaptic glutamatergic circuitry, in which the entorhinal cortex is the major source of input. Information flow through the hippocampus is initiated in the dentate gyrus (DG) and proceeds to CA3 and then, CA1. In turn, CA1 and subiculum send efferent back to the deep layers of the entorhinal cortex. While this classic tri-synaptic network participates in memory and learning processes, current evidence reveals an alternative circuitry in which DG neurons also project to CA2, which in turn connects to CA1 pyramidal cells (Kohara et al., 2014). Interestingly, this circuitry establishes a direct connection between DH and VH, supporting a pathway to modulate brain areas linked to ventral CA1; for instance, prefrontal cortex and basolateral amygdala (Kohara et al., 2014). Moreover, behavioral studies have revealed that CA2 circuits are crucial for social (Sotomayor-Zárate et al., 2010), temporal (Mankin et al., 2015) and probably emotional aspects of memory.

Disturbances in glutamate-mediated neurotransmission have been associated with several diseases, including cognitive impairment, anxiety and mood disorders (Sanacora et al., 2012). Hippocampal glutamatergic neurotransmission is significantly affected in animals exposed to stress, as well as in depressed patients (Sanacora et al., 2012). For instance, rodents exposed to a stressor show increased glutamate release in the hippocampus (Moghaddam et al., 1994; Popoli et al., 2011). Nonetheless, few studies have evaluated the possible differences in the response of DH and VH to stressful stimuli. Exposure of rats to a chronic unpredictable stress paradigm produces opposed effects in hippocampal poles. In the VH, stress promotes an increase in its volume, concomitant with an increase in the length of CA3 apical dendrites (Pinto et al., 2015). In contrast, in the DH, stress triggers a reduction of hippocampal volume as a consequence of decreased length of apical dendrites of CA3 and CA1 neurons (Pinto et al., 2015) and reduced dendritic spine density (Castañeda et al., 2015). Additionally, chronic stress impairs spatial memory (Pinto et al., 2015) and induces anxiety-like behaviors (Ulloa et al., 2010). These evidences suggest that stress-induced dendritic length variations have functional consequences in both DH and VH. The structural modifications observed under chronic stress in DH and VH may not only imply a variation in the number of excitatory synapses, but also in the abundance of glutamatergic receptors. The α-amino-3-hydroxy-5-methyl-4-isoxazole propionic acid receptor (AMPAR) is an ionotropic glutamate receptor that typically produces a fast excitatory synaptic depolarization (Anggono and Huganir, 2012; Henley and Wilkinson, 2016). The AMPAR has four different subunits (GluA1–GluA4), conforming a heterotetramer, and is organized as a dimer of dimers (Anggono and Huganir, 2012; Henley and Wilkinson, 2016). Studies have demonstrated that pyramidal neurons of the hippocampus have higher expression levels of GluA1 and GluA2 than GluA3 and GluA4 subunits (Geiger et al., 1995). Furthermore, CA1/CA2 pyramidal neurons express AMPAR complexes composed by GluA1/GluA2 and GluA2/GluA3 subunits (Wenthold et al., 1996). The subunit composition of AMPARs not only modulates cation permeability (Na^+^ vs. Ca^2+^; Anggono and Huganir, 2012; Henley and Wilkinson, 2016), but also modulates activity-dependent potentiation of synaptic transmission, such as long-term potentiation (LTP; Yang et al., 2010; Selcher et al., 2012). The N-methyl-D-aspartate receptor (NMDAR) is highly expressed in the hippocampus; it has a dual ligand and voltage gating, and mediates Na^+^, K^+^ and Ca^2+^ conductance (Flores-Soto et al., 2012; Sanz-Clemente et al., 2013). There are seven different NMDAR subunits: NR1, NR2 (A, B, C and D) and NR3 (NR3A and NR3B), which form a heterotetramer conformed by dimer of dimers (Flores-Soto et al., 2012; Sanz-Clemente et al., 2013). Each dimer contains the mandatory presence of NR1, which binds glycine a co-agonist and assembles with NR2 or NR3 subunits (Fukaya et al., 2003). Receptors conformed by NR1/NR2A and NR1/NR2B subunits have different electrophysiological properties and synaptic location (Sanz-Clemente et al., 2013). Although several studies have focused on the anatomic and functional differences between DH and VH, few studies have described the effect of chronic stress in the relative abundance and subunit composition of AMPAR and NMDAR. For instance, chronic unpredictable stress in adult rats elicits increased expression of the obligatory NR1 subunit, as well as of the accessory subunits NR2A and NR2B, at both mRNA and protein levels, and mainly in the VH; however, variations in AMPARs subunits were not explored (Calabrese et al., 2012).

Considering that chronic restraint stress induces cognitive alterations, anhedonia, depression-like and anxiety-like behaviors, we explored whether chronic stress modifies the expression of c-Fos and Arc immediately early genes (IEG) and both NMDA and AMPAR subunits differently in DH and VH, along with PSD-95, which is a neuronal PDZ protein that associates with receptors and cytoskeletal elements at the synapse. We also evaluated whether stress triggers variations in their relative abundance in synaptosomes (fraction composed of a resealed presynaptic terminal and postsynaptic density components). The present work shows that stress affects the DH and VH differently by promoting specific variations in the levels of AMPAR and NMDAR subunits. These findings may contribute to expand our understanding of the plethora of neuroplastic events triggered by chronic stress and may explain the contrasting effect in the functioning of both hippocampal poles; i.e., impairment in cognitive function and exacerbation of emotional response observed under chronic stress.

## Materials and Methods

### Animals

Male Sprague-Dawley rats used in these experiments were obtained from the Faculty of Chemical and Pharmaceutical Sciences, Universidad de Chile. Efforts were made to minimize both the number of animals used and their suffering. The rats were handled according to guidelines outlined and approved by the Ethical Committee of the Faculty of Chemical and Pharmaceutical Sciences (CBE N°2011-7-4, 2012), Universidad de Chile, and the Science and Technology National Commission (CONICYT), in compliance with the National Institutes of Health Guide for Care and Use of Laboratory Animals (NIH Publication, 8th Edition, 2011).

Rats were handled once per day for a week, prior to the experimental procedures. The handling procedure consisted in picking up the rat by its body, weighing it and returning it to its home cage. Rats were randomly assigned to two weight-matched groups: unstressed animals (control group, *n* = 15) and restraint stressed animals (stress group, *n* = 16). All procedures were carried out during the light phase of the light-dark cycle. The restraint stress procedure was carried out between 9:00 and 12:00, as previously described (Bravo et al., 2009; Ulloa et al., 2010; Castañeda et al., 2015; García-Rojo et al., 2017). Briefly, rats were placed in a transparent plexiglass tube (25 × 8 cm) during 2.5 h for 14 consecutive days. After restraint, rats were returned to their home cages. Unstressed animals were left undisturbed in their home cages. Twenty-four hours after the last stress session, the animals were decapitated. In order to evaluate whether some stress-induced changes (c-Fos and Arc mRNA and protein levels) were associated with an adaptive response to chronic stress exposure rather than an acute effect of daily stress exposure, a group of acutely stressed animals was included. For this purpose, animals were restrained for 0.5 h (*n* = 3) or 2.5 h (*n* = 4) and immediately sacrificed. To evaluate post-stress effects, another group of animals was stressed during 2.5 h and then sacrificed 24 h after stress exposure (*n* = 4). A sample of trunk blood was taken for corticosterone (CORT) determination.

Brains were rapidly obtained and dorsal and ventral portions of hippocampus were defined by their position relative to Bregma, according to Paxinos coordinates (Paxinos, 1982). In brief, hippocampi were quickly placed over a cooled Petri dish and dissected the top 2/5 as DH, the bottom 2/5 as VH and the middle portion was discarded. The tissues were then frozen under liquid N_2_ and stored at −80°C until processing for RNA and protein level determinations by RT-qPCR or western blot, respectively. The tissues obtained from one hemisphere were used for RNA isolation and the other one was used for protein extraction.

### Fecal Pellet Output

The number of pellets emitted by the animals during the restraint stress session was monitored at the end of each stressor exposure (i.e., after acute exposure or immediately after each of the 14 exposures of the chronic model). Stressed animals were then immediately sacrificed (acute) or placed in a cage with clean bedding (chronic group). To obtain an estimation of fecal output in controls and considering that animal isolation may induce a stress response, control animals were kept in a group of 2–3 animals and the cage bedding was changed every day. At this moment, the fecal pellet output was determined after 2.5 h each day during 14 consecutive days. Thus, the fecal output of control animals represents the mean value for a group. For comparison with acutely stressed animals (0.5 h), an extrapolation of fecal pellet output in control animals was also carried out at 0.5 h.

### Serum Corticosterone Determination

Trunk blood samples were collected for the determination of serum CORT levels. Special care was taken to avoid pre-decapitation stress while decapitation took place, so the other animals were left outside the room during this process. Blood was then centrifuged at 4000× *g* for 15 min, and serum was collected and stored at −20°C. Hormone level determination was carried out using CORT ELISA Kit (Enzo, New York, NY, USA; Cat. ADI-900-097), according to the instructions provided by the kit.

### RT-qPCR

RNA was extracted from dorsal and ventral hippocampi with miRNeasy kit (Qiagen, Hilden, Germany), according to the manufacturer’s instructions. The concentration of each RNA sample was determined by Nanodrop 2000 (Thermo Scientific, USA) and integrity was evaluated by denaturing gel electrophoresis. RNA was reverse transcribed into cDNA by using Superscript II (Invitrogen, Carlsbad, CA, USA) following the manufacturer’s instructions and as was reported previously (Castañeda et al., 2015). RT-qPCR experiments were conducted on the Stratagene Mx3000p thermocycler (Stratagene, Agilent), programmed as follows: 95°C for 10 min followed by 40 cycles of 95°C for 15 s, 60°C for 15 s and 72°C for 20 s. Each reaction was carried out in duplicate and consisted of 10 μl Brilliant II Ultra-fast SYBR Green QPCR Master Mix (Agilent Technologies), an appropriate dilution of RT and 0.12 μM of the forward and reverse primers designed using Primer-Blast, NCBI and obtained from Integrated DNA Technologies (Coralville, IA, USA). The sequence of primers is shown in Table 1. Standard curves for all primer sets were conducted with serial dilutions of cDNAs, and specificity was validated using melting curve analyses. Relative gene mRNA levels were calculated based on the 2^−ΔΔCT^ for each mRNA, and normalized to that of the β-actin housekeeping gene mRNA.

**Table 1:** Primer sequences.

Transcript	Forward primer	Reverse primer	Amplicon size	Accession N°
c-Fos	5′-GTTTCAACGCGGACTACGAG-3′	5′-GGCACTAGAGACGGACAGAT-3	161 bp	NM_022197.2
Arc	5′-GCATCTGTTGACCGAAGTGTCC-3′	5′-GCACCCAAGACTGGTATTGCTG-3′	455 bp	NM_019361.1
NR1	5′-GATGTCTTCTAAGTATGCGGACGG-3′	5′-TCACTCATTGTGGGCTTGACG-3′	276 bp	NM_001270610.1
NR2A	5′-CCCTGCACCAATTCATGGTC-3′	5′-AGGTGGTTGTCATCTGGCTC-3′	220 bp	NM_012573.3
NR2B	5′-AACAGGTGCCTAGCCGATG-3′	5′-CAAAGAAGGCCCACACTGAC-3′	160 bp	NM_012574.1
GluA1	5′-GGAAGGAAGGGAGGAAGGAAAG-3′	5′-GGAGAACTGGGAACAGAAACGG-3′	383 bp	NM_031608.1
GluA2	5′-CTACCGCAGAAGGAGTAGCC-3′	5-TTACTTCCCGAGTCCTTGGC-3′	289 pb	NM_017261.2
Actin-β	5′-TTGTCCCTGTATGCCTCTGGTC-3′	5′-ACCGCTCATTGCCGATAGTG-3′	346 bp	NM_031144.3

### Total and Synaptic Protein Preparation

Dorsal and ventral hippocampi were slowly thawed in cold homogenization buffer that contained: 0.32 M sucrose, 1 mM EDTA, 1 mM EGTA, 10 mM HEPES, 5X protease inhibitor (cOmpleteTM EDTA-free, Sigma-Aldrich) and 1X phosphatase inhibitor (PhosStopTM, Sigma Aldrich) at pH 7.4. Samples were homogenized in glass-teflon homogenizer (15 strokes) and then centrifuged at 430× *g* for 10 min at 4°C. Supernatant was kept on ice and a fraction was taken (representing the total homogenized fraction or H fraction) and the pellet was re-homogenized in buffer and centrifuged again at 600× *g* for 10 min at 4°C. The pellet (corresponding to the nuclear fraction or N fraction) was then mixed with the lysis buffer: 150 mM NaCl, 50 mM Tris, 1 mM EDTA, 1 mM EGTA, 1% Triton X-100, 1% deoxycholate, 5X protease inhibitor and 1X phosphatase inhibitor, at pH 7.4. On the other hand, both supernatants were mixed and centrifuged at 20,000× *g* for 45 min at 4°C, and the pellet (corresponding to the synaptosomal fraction or S fraction) was minced and resuspended with lysis buffer. Each protein fraction was quantified with bicinchoninic acid (BCA; Pierce™ BCA Protein Assay Kit Thermo Fisher Scientific, MA, USA). Samples were mixed with loading buffer that contained: 250 mM Tris, 5% SDS, 6.7% v/v glycerol, 0.5 mg/mL DTT and 13.3 mg/mL bromophenol blue at pH 6.8. Finally, the samples were boiled for 2 × 5 min at 100°C and stored at −80°C for western blot evaluation.

### Electron Microscopy

In order to characterize the synaptosomal fraction, an aliquot was fixed overnight with glutaraldehyde 2.5% in sodium cacodilate buffer (0.1 M, pH 7.0). The pellet was washed with the same buffer three times during 2 h. Samples were post fixed with 1% osmium tetroxide during 90 min and then washed in double distilled water. Samples were stained with 1% uranile acetate during 1 h. Afterwards, samples were dehydrated in a battery of acetone solutions, increasing acetone concentration (50, 70, 95 and 100%). Samples were embedded in Epon:acetone (1:1) overnight, then placed in fresh pure Epon resin and allowed to polymerize at 60°C during 48 h. Thin sections (60 nm) obtained in an ultramicrotome Sorvall MT5000 were stained with uranile acetate (4% methanol) during 2 min and finally stained with lead citrate during 5 min. Observations were performed on a Philips Tecnai 12 Biotwin electron microscope (Eindhoven, Netherlands), at 80 kV.

### Semi-Quantitative Western Blotting

A total of 15 μg (synaptosomes) or 30 μg (total fraction) of proteins were resolved on 8% SDS-polyacrylamide gels and then blotted onto 0.2 μm nitrocellulose (for detection of all NMDAR and AMPAR subunits and PSD-95) or 0.2 μm PVDF membranes (for determination of β-actin, α-tubulin and lamina-associated polypeptide (LAP)-2a). Membranes were blocked in different solutions and were then incubated with the proper primary antibody diluted in blocking solution (Table 2). The blots were rinsed with PBS 1X or TBS 1X-0.1% Tween-20 (TBS-T), according to the specific antibody, and were incubated at room temperature for 2 h with peroxidase-conjugated anti-rabbit or anti-mouse secondary antibody (1:10,000, Calbiochem, Cambridge, MA, USA). All membranes were then incubated with enhanced chemiluminescent (ECL) substrate (Perkin Elmer Life Sciences, Boston, MA, USA) and were detected by a chemiluminescence imager (Syngene, UK). After that, blots were stripped with ReBlotPlus Mild Antibody Stripping Solution (Sigma-Aldrich, St. Louis, MO, USA) during 10 min and after several washes in PBS each membrane was reprobed with antibody against the protein selected as loading control. The levels of β-actin and α-tubulin were used as protein loading control of total hippocampal and synaptosomal fractions, respectively. Band intensities were determined and analyzed using the UN SCAN IT software (RRID: SCR_013725).

### Immunofluorescence Staining

Animals were anesthetized with isofluorane (1.5% v/v air) as we have previously described (Bravo et al., 2009) and then perfused intracardially with heparinized (2 UI/mL) saline followed by PFA 4% in PBS. In these animals, it was not possible to obtain blood samples for CORT determination. Brains were post-fixed in the same solution for 24 h, which was then replaced with 30% p/v sucrose-PBS. The fixed brains were quick-frozen in isopentane cooled by liquid nitrogen for 30 s. The brains embedded in OCT compound were cut with a cryostat (HM500 O; Microm, Heidelberg, Germany) on the coronal plane at 30 μm thicknesses as follows: DH Bregma −2.3 to −4.0 and VH −4.5 to −5.80, according to the Paxinos Atlas (Paxinos, 1982). Slices were stored in a cryoprotectant solution (30% ethylene glycol, 30% glycerol, 10% 0.2 M sodium phosphate buffer pH 7.4) at −20°C. In the case of animals stressed acutely, frozen un-fixed brain was cutted and then fixed in 4% PFA in PBS during 10 min, and washed several times. Slices were then processed for immunofluorescence, as previously reported, with some modifications (Rojas et al., 2014). In brief, brain slices were permeabilized with 0.1% Triton-X100 in PHEM buffer (PHEM: 60 mM PIPES, 25 mM HEPES, 5 mM EGTA, 1 mM MgCl_2_, pH 6.9) for 15 min and then incubated with 50 mM NH_4_Cl for 5 min. After washing, slices were incubated in blocking solution (1% FBS, 2% BSA, 0.1% fish gelatin in PHEM) for 1 h and then incubated overnight with appropriate dilution of antibodies, as described in Table 2 (1:500 for NR1, NR2A, NR2B and 1:1000 for Arc), in blocking solution. The detection of MAP2a was carried out with a 1:5000 dilution of mouse monoclonal antibody (M4403, Sigma-Aldrich, St. Louis, MO, USA). The detection of c-Fos was based on a described protocol (Sotomayor-Zárate et al., 2010), with slight modifications. We used simultaneous blocking/permeabilization during 1 h (blocking solution, 0.2% Triton-X100) and then the slices were incubated overnight with a 1:500 dilution of rabbit polyclonal antibody (sc7202, clone H125, Santa Cruz Biotechnology, Dallas, TX, USA) in blocking solution. After three washes with PHEM buffer, samples were incubated overnight at 4°C with Alexa Fluor 488-conjugated goat anti-mouse IgG (1:500; Molecular Probes, Eugene, OR, USA) to detect NR1 subunit and MAP2a, and with Alexa Fluor 568-conjugated goat anti-rabbit IgG (1:500; Molecular Probes, Eugene, OR, USA) to detect NR2A, NR2B, c-Fos and Arc. Finally, all sections were counterstained with Hoechst 33342 (0.5 μg/ml in PBS) and mounted with DAKO fluorescent mounting Medium (DAKO, Carpinteria, CA, USA). Brain sections from control and stressed animals were stained simultaneously to ensure uniform conditions for subsequent quantitative analysis. Brain slices were photographed in Zeiss LSM 700 confocal laser scanning microscope (Oberkochen, Germany). Confocal z-stacks separated by a z-step of 4 μm were captured using a Plan-Apochromat 10× (0.3 NA) and 40× (1.4 NA) Zeiss oil-immersion objective. Settings for pinhole size, aperture gain, and offset were initially adjusted and then held constant to ensure that all images were digitized under identical resolution and the emission of both fluorophores were acquired separately. Negative controls were obtained in the absence of primary or secondary antibody. To allow direct comparison of different data sets, imaging was performed using the same parameters for laser excitation (i.e., intensity) and photomultiplier tube gain (i.e., sensitivity) in each set of samples. All confocal images were processed using ImageJ US NIH^1^ tools. For immunofluorescence quantification, several sectors of hippocampus were selected for analysis *(stratum radiatum* (SR), *stratum lacunosum moleculare* (SLM) and molecular layer (ML)). The mean immunofluorescence intensities were measured by one experimenter blinded to the animal condition.

**Table 2:** Primary antibodies and blocking conditions.

Antigen	Immunogen	Source, Host species, Cat#, RRID	Concentration used	Blocking solution
β-Actin	Synthetic actin N-terminal aa. DDDIAALVIDNGSGK	Sigma-Aldrich, mouse monoclonal, Cat# A5316, RRID:AB_476743	0.1 ng/μL	3% non-fat milk-TBS 0.1% Tween-20
α-Tubulin	Purified chick brain tubulin	Sigma-Aldrich, mouse monoclonal, cat# T9026, RRID:AB_477593	0.1 ng/μL	3% non-fat milk-TBS 0.1% Tween-21
GluA1	Synthetic peptide (aa 895-907 in rat GluA1) coupled to hemocyanin	Synaptic Systems, mouse monoclonal, cat# 182 011, RRID:AB_2113443	1 ng/μL	1% BSA-PBS 1X
GluA2	Recombinant protein of rat GluA2 (C terminus; aa 836–883)	Synaptic Systems, rabbit polyclonal, cat# 182 103, RRID:AB_21113732	0.5 ng/μL	3% non-fat milk-PBS 1X
LAP-2a	Rat LAP2 aa. 34–156	BD Biosciences, mouse monoclonal, Cat# 611000, RRID:AB_398313	0.5 μg/μL	3% non-fat milk-TBS 0.1% Tween-20
NR1	Recombinant protein of NR1 (aa 660–811)	Synaptic Systems, mouse monoclonal, cat# 114 011, RRID:AB_887750	1 ng/μL	3% non-fat milk-PBS 1X
NR2A	6 His-tagged fusion protein corresponding to aa 1265–1464 of mouse NR2A	Millipore, rabbit polyclonal, cat# 07-632, RRID:AB_310837	2 ng/μL	1% BSA-PBS 1X
NR2B	Synthetic peptide (aa 42–60 of rat NR2B) coupled to hemocyanin	Synaptic Systems, rabbit polyclonal, cat# 244 103, RRID:AB_10805405	0.5 ng/μL	1% BSA-PBS 1X
PSD-95	Synthetic peptide of residues 50–150 of mouse PSD-95 conjugated to KLH	Abcam, rabbit polyclonal, Cat# ab18258, RRID:AB_444362	0.5 μg/μL	0.5% BSA PBS

### Statistical Analysis

All values are expressed as mean ± SEM. All data were analyzed comparing two groups (control and stress) in dorsal or VH. When it was not possible to conduct a normality test distribution, normal data distribution was not assumed. Thus, differences between two groups were analyzed with *t*-test or Mann-Whitney *U* test (two-tail and in some cases one-tail based in previous data, suggesting that the difference, if any, can only go in one direction). We used the Kruskal-Wallis test for comparison of three or more groups, followed by Dunn’s *post hoc* test. *p* < 0.05 was considered significant. Graphpad Prism (RRID: rid_000081; Graphpad Software, San Diego, CA, USA) was used for statistical analysis.

## Results

### Effect of Repeated Restraint on Body Weight Gain, Fecal Output during Stress Procedure and Adrenal Gland Weight

**Figure 1:**
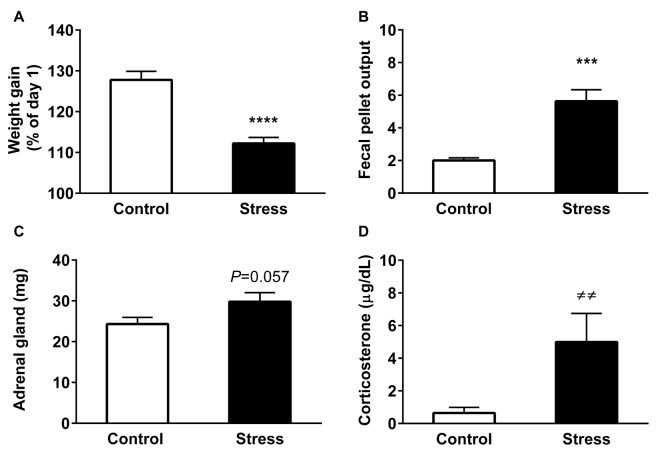
Chronic restraint stress modifies physiological parameters. All graphs represent mean ± SEM of each group. **(A)** Variation of body weight gain was evaluated at the end point of treatment. All rats were weighed daily and one group was stressed during 14 consecutive days. **(B)** Fecal output was determined every day in control (2.5 h) and stressed animals during the stress session. Data represent the mean value/day. **(C)** Adrenal gland weight of control and stressed animals **(D)** Serum corticosterone (CORT) levels in control and stressed animals. Data represent mean ± SEM. Control (*n* = 15), Stress (*n* = 16). Data have a normal distribution with the exception of CORT levels. The former were analyzed by two-tailed *t*-test, ****P < 0.0001, ***P < 0.001, *t*-test two-tail. Differences in CORT levels were analyzed by two-tailed Mann-Whitney *U* test, ^≠≠^*P* = 0.0018.

The body weight gain of the rats at the experimental end-point is depicted in Figure 1A. Analysis revealed that control animals increased their initial weight by 30%; in contrast stressed animals gained approximately 10% (*P* < 0.0001). Additionally, we evaluated the effectiveness of the restraint procedure by measuring fecal output every day during the stress procedure. This stress response is driven by corticotropin-releasing hormone (CRH), producing an activation of colon motility (Nakade et al., 2007a,b). We found a significant increase in the number of feces pellets in stressed animals, compared to unstressed animals (Figure 1B, *P* < 0.0001). We also determined whether restraint stress induced variations in adrenal gland weight and CORT levels, as an index of hypothalamic-pituitary-adrenal (HPA) activation. The weight of adrenal glands tended to increase in stressed animals (Figure 1C, *P* = 0.057), a variation that correlates with a rise in serum CORT levels measured in basal conditions (blood sampled between 9:30 and 11:00 AM; Figure 1D, *P* = 0.0001). Overall, these results demonstrate the activation of the HPA axis, suggesting that animals did not adapt to daily exposure to the homotypic stressor.

### Stress Affects mRNA Levels of Immediate Early Genes in Dorsal and Ventral Hippocampus

**Figure 2:**
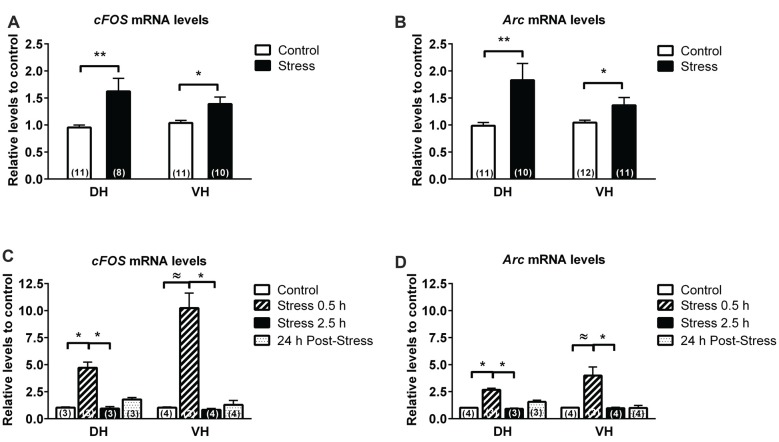
Comparison in the expression of immediate early genes (IEG) in dorsal (DH) and ventral hippocampus (VH) of chronically and acutely stressed animals. RT-qPCR analysis of *Fos* **(A,C)** and *Arc* **(B,D)**, evaluated in DH and VH of control and stressed animals. Data were analyzed by 2^−ΔΔCt^ using *β-actin* as normalizer. Bar graphs represent mean ± SEM of each group. Number inside bars represent N° of animals. Differences between control and stressed animals were determined by two-tail Mann-Whitney test. **P < 0.01; *P < 0.05. Differences in control and acutely stressed animals were evaluated with Kruskal-Wallis test and Dunn’s *post hoc* test, *P < 0.05. Two-tail Mann-Whitney analysis was used to compare the effect of 0.5 h of stress with control. ^≈^*P* < 0.05.

We detected, 24 h after the last session of stress, a 60% raise in c-fos mRNA in DH (*P* < 0.01) and 40% raise in VH (*P* < 0.05; Figure 2A). We found that expression of activity-regulated cytoskeleton-associated protein (Arc) mRNA—a marker of neuronal activity—was also low in both hippocampal poles, but was increased by 80% (*P* < 0.01) and 40% (*P* < 0.05) in DH and VH, respectively (Figure 2B).

To evaluate whether these changes corresponded to an adaptive response of chronic stress rather than acute response to daily restraint exposure, we included a group of acutely stressed animals. This group of animals was stressed during 0.5 h or 2.5 h and sacrificed immediately. During the stress session, animals showed an increase of feces output relative to control (stress 0.5 h, 9.3 ± 1.8 feces; stress 2.5 h, 9 ± 1.9 feces; control 0.5 ± 0.1 feces). Analysis of the variation in CORT levels by Kruskal Wallis indicated differences between treatments (*P* < 0.0001) and Dunn’s post-test indicated that stressed animals during 2.5 h (35.8 ± 9.9 μg/dL; *P* < 0.01), but not during 0.5 h (10.15 ± 1.86 μg/dL), showed a significant rise in CORT in comparison to control (0.98 ± 1.2 μg/dL). Furthermore, 24 h after a single stress of 2.5 h, animals showed similar basal CORT levels (5.45 ± 2.36 μg/dL), compared to control. Kruskal Wallis analysis of c-Fos mRNA levels indicated differences between groups (*P* = 0.001) and Dunn’s post-analysis revealed an increase of this transcript in DH after 0.5 h of restraint (i.e., 370% over control, *P* < 0.05) and a significant reduction after 2.5 h of stress (*P* < 0.05; Figure 2C). In addition, 24 h after stress, c-Fos mRNA levels did not differ to control or 0.5 h stressed animals. The VH showed high sensitivity to stress, displaying an increment of c-Fos mRNA almost 900% over control; Kruskal Wallis analysis indicated differences between groups (*P* = 0.039) and Dunn’s analysis revealed a significant difference between groups of stressed animals (Figure 2C). Nonetheless, with Mann-Whitney *U*-test we detected a significant difference between control and the group of animals stressed during 0.5 h (*P* < 0.05). Furthermore, when we evaluated the effect of acute stress on Arc mRNA levels, the Kruskal Wallis analysis indicated differences between groups both in DH (*P* = 0.002) and VH (*P* < 0.05). *Post hoc* Dunn’s analysis indicated that 0.5 h of stress causes a significant increase of 150% over control in DH (*P* = 0.05), value that returned to basal levels after 2.5 h of stress (Figure 2D). The response of VH to acute brief stress exposure (0.5 h) triggers a significant rise in comparison to animals stressed for 2.5 h (*P* = 0.05); variation which was only significant compared to control groups using the Mann-Whitney *U* test (Figure 2D).

### Effect of Stress of c-Fos and Arc Protein Levels in DH and VH

**Figure 3:**
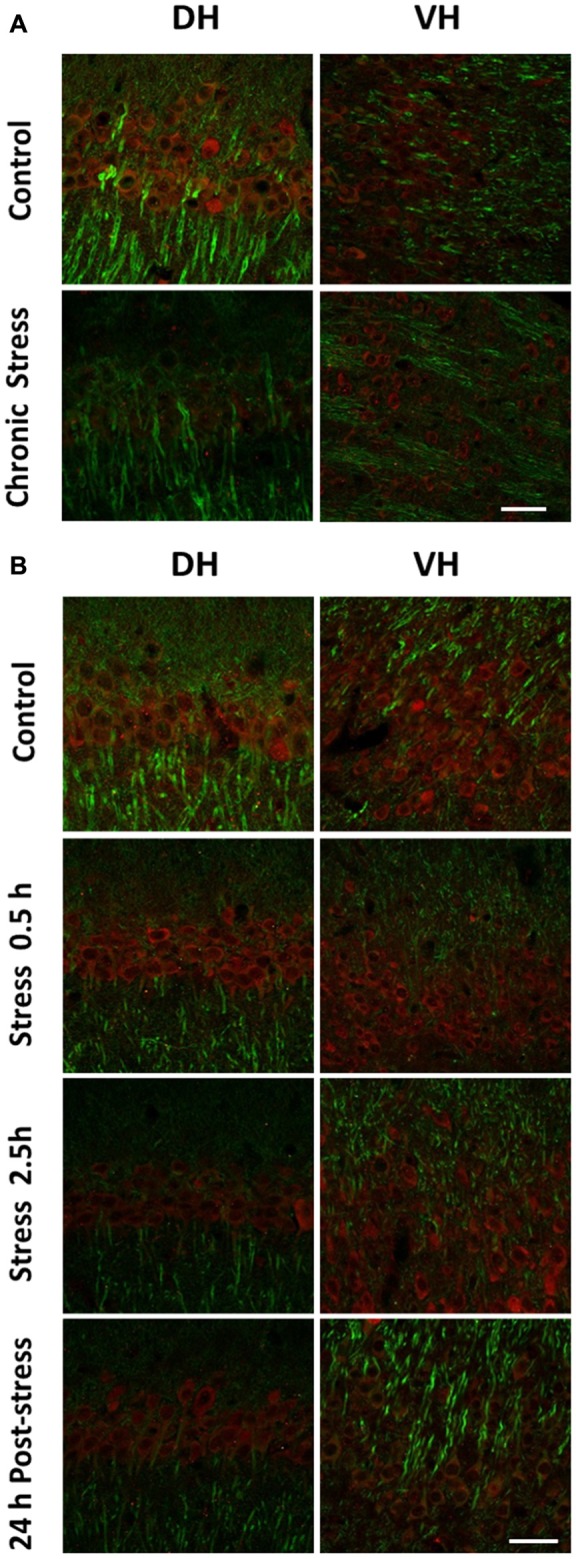
Comparison in the levels c-Fos in DH and VH of chronically and acutely stressed animals. Immunoreactivity for c-Fos was evaluated in DH and VH of control (*n* = 4) and stressed animals during 14 days. **(A)** Panel shows a representative confocal microscopic images displaying immunoreactivity of c-Fos (red) and MAP2a (green) in CA1 field from DH and VH of control and stressed animals. **(B)** Panel shows a representative confocal microscopic images displaying immunoreactivity of c-Fos (red) and MAP2a (green) obtained from DH and VH of control (*n* = 3), stressed animals (0.5 h, *n* = 3 and 2.5 h, *n* = 3) or 24 h after a single stress session of 2.5 h (*n* = 3). Scale bar = 200 μm.

We decided to examine variations of IEG protein levels in brain slices of stressed animals by using indirect immunofluorescence. Representative image of CA1 area of chronically stressed animals and control is shown in Figure 3A. A low immunoreactivity for c-Fos was observed in both DH and VH of control and stressed animals (Figure 3A). In acutely stressed animals, we only detected a slight increase in c-Fos immunoreactivity at 2.5 h of stress in CA1 area (Figure 3B). Considering the low immunoreactivity detected, it was not possible to conduct semiquantitative analysis.

**Figure 4:**
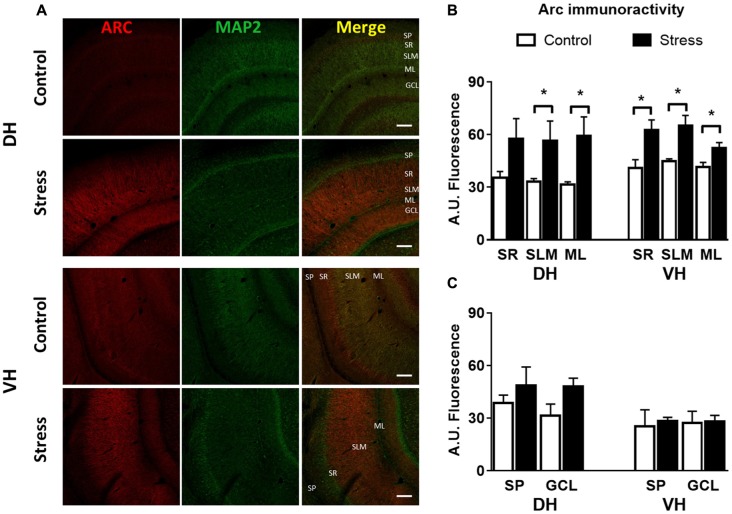
Chronic stress produces an enhancement in Arc immunoreactivity at synaptic layers of DH and VH. **(A)** Confocal microscopic images displaying immunoreactivity of Arc (red) and MAP2a (green) in DH and VH of control and chronically stressed animals. Scale bar = 200 μm. Quantification of the immunoreactivity detected at the dendritic **(B)** and somatic **(C)** layers of both DH and VH. SP, *stratum pyramdalis*; SR, *stratum radiatum*; SLM, *stratum lacunosum moleculare*; ML, molecular layer; GCL, granular cell layer. Graphs represent mean ± SEM of each group. Differences between control and stressed animals were determined by two-tail Mann-Whitney test. *P < 0.05.

**Figure 5:**
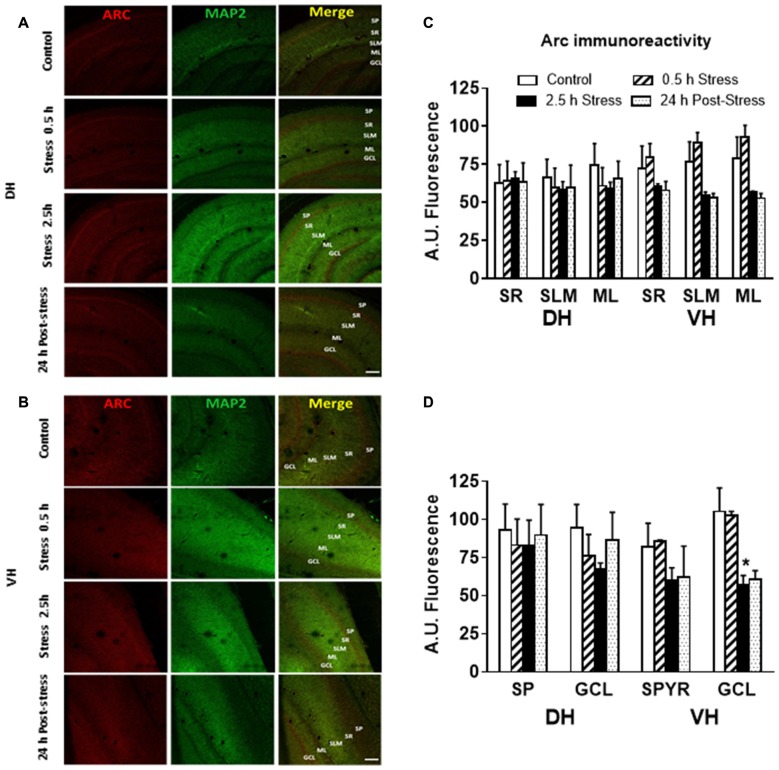
Effect of acute stress in Arc immunoreactivity at synaptic and somatic layers of DH and VH. **(A)** Confocal microscopic images displaying immunoreactivity of Arc (red) and MAP2a (green) in DH **(A)** and VH **(B)** of control and chronically stressed animals. Scale bar = 200 μm. Quantification of the immunoreactivity detected at the dendritic **(C)** and somatic **(D)** layers of both DH and VH. SP, *stratum pyramdalis*; SR, *stratum radiatum*; SLM, *stratum lacunosum moleculare*; ML, molecular layer; GCL, granular cell layer. Graphs represent mean ± SEM of each group, *n* = 3–4 animals. Differences in control and acutely stressed animals were evaluated with Kruskal-Wallis test and Dunn’s *post hoc* test. Two-tail Mann-Whitney analysis was used to compare the 0.5 h of stress with 2.5 h of stress. *P < 0.05.

Additionally, immunofluorescence analysis of Arc in brain slices of chronically stressed animals revealed an increase in the immunoreactivity at the neuropil area of DH and VH (Figure 4A). Quantitative analysis showed a significant increase in Arc immunoreactivity in the *stratum lacunosum moleculare* (SLM; *P* < 0.05) and molecular layer (ML; *P* < 0.05) in both DH and VH (Figure 4B). Furthermore, a rise in Arc immunoreactivity was detected in the *stratum radiatum (SR)* of VH (Figure 4B). Furthermore, no changes in immunoreactivity for Arc were observed at *stratum pyramidalis* (SP) and granular cell layer (GCL) of both areas (Figure 4C). In contrast to these findings, acute stress did not modify the immunoreactivity at dendritic layers of both DH and VH (Figures 5A–C) Nonetheless, 2.5 h of stress promoted a significant reduction of Arc immunoreactivity at the GCL of VH (Figure 5D).

### Stress Induces Differential Expression of AMPAR Subunits in Dorsal and Ventral Hippocampus

**Figure 6:**
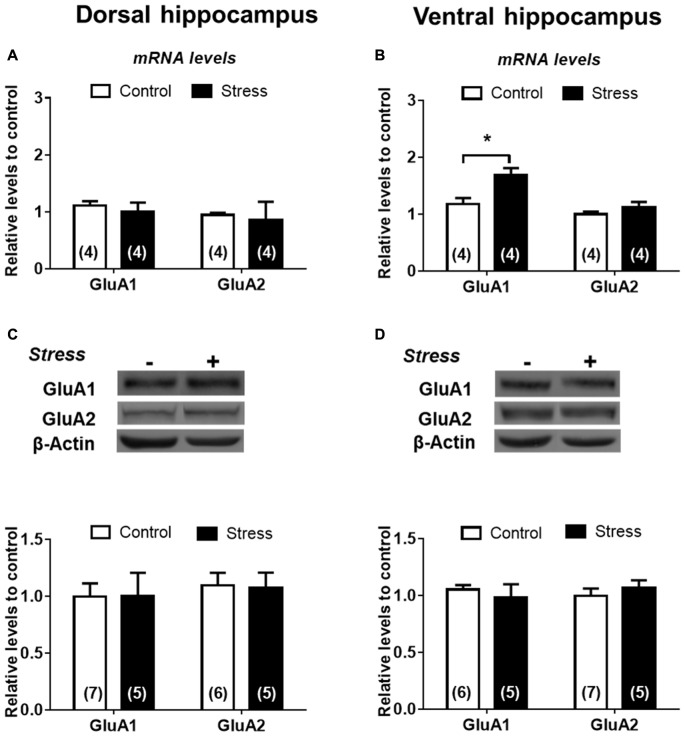
α-amino-3-hydroxy-5-methyl-4-isoxazolepropionic acid (AMPA) subunits are differentially expressed in DH and VH in chronically stressed animals. Evaluation of GluA1 and GluA2 mRNA levels in dorsal **(A)** and ventral **(B)** hippocampus. Data were analyzed by 2^−ΔΔCt^ using *β-actin* as normalizer. Levels of GluA1 and GluA2 protein levels from total extract of dorsal **(C)** and ventral **(D)** hippocampus. Graphs represent mean ± SEM of each group. Number of animals is inside bars. *P < 0.05; two-tail Mann-Whitney test.

**Figure 7:**
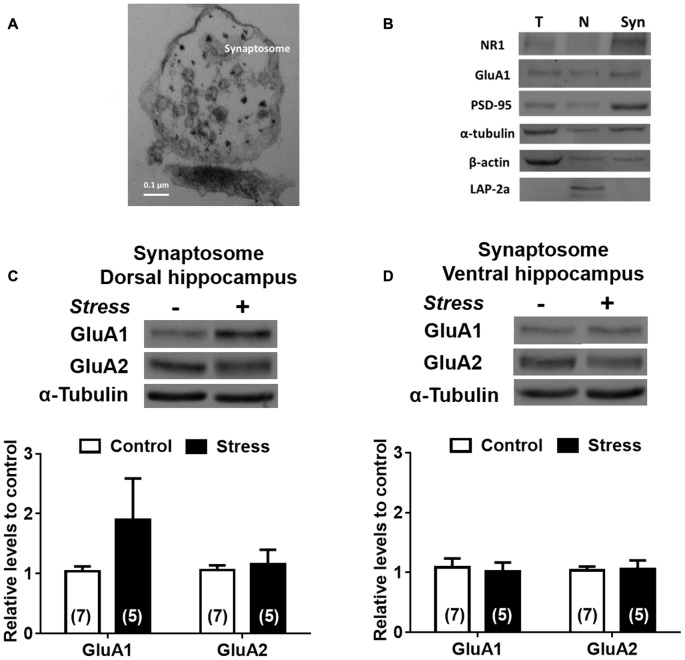
Effect of chronic stress on AMPA subunit levels in synaptosomal fraction of DH and VH. **(A)** Morphological characterization of synaptosomal fraction by electron microscopy. Photograph of the synaptosomal fraction; shows the presynaptic button and the presynaptic vesicles attached at the postsynaptic density. Picture was taken with 105,000× magnification. **(B)** Biochemical characterization of the different fractions obtained during synaptosome preparation. H represents the hippocampal homogenate, N represents nuclear fraction and Syn represents the synaptosomal fraction. Markers were evaluated by immunoblot. The nuclear marker (LAP-2a) was not detected in synaptosomal fraction. The PSD-95 (Post-synaptic marker), GluA1 and NR1 subunits of AMPA and NMDA receptor (NMDAR), respectively were enriched at the synaptosomal fraction. Levels of GluA1 and GluA2 subunit were evaluated in synaptosomal fraction obtained from dorsal** (C)** and ventral **(D)** hippocampus. Upper panel shows a representative western blot. Graphs represent mean ± SEM of each group. Number of animals is inside bars.

To examine the effect of chronic stress on glutamatergic transmission, we first determined pattern expression of AMPAR subunits and although chronic stress did not promote variations of GluA1 and GluA2 mRNAs levels in DH (Figure 6A) it did increase the levels of GluA1 mRNA in VH (Figure 6B). Furthermore, GluA1 and GluA2 protein levels in both whole extracts were not modified by chronic stress (Figures 6C,D). To examine whether chronic stress promoted changes in the distribution of receptor subunits at synaptic sites, we prepared a synaptosomal fraction from DH and VH. By electron microscopy, we confirmed that this fraction is enriched in axon terminals (containing vesicular structures) and adherent postsynaptic densities, which evidences intact synapses (Figure 7A). Western blotting analysis revealed that lamina-associated polypeptide (LAP) 2a, which corresponds to a nuclear marker, is not detected in this fraction (Figure 7B). Furthermore, the synaptosomal fraction displayed an enrichment of post-synaptic proteins, such as the PSD-95, NR1 and GluA1 subunits of NMDAR and AMPAR respectively (Figure 7B). In synaptosomes obtained from both areas, we observed that levels of GluA1 and GluA2 are insensitive to stress (Figures 7C,D).

### Stress Differentially Modifies the Expression of NMDAR Subunits in Dorsal and Ventral Hippocampus

**Figure 8:**
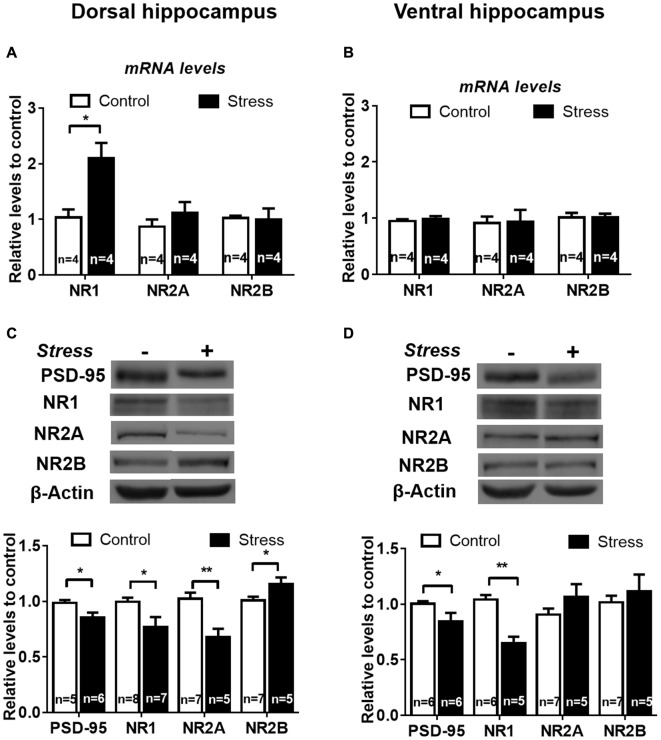
Chronic stress promotes differential expression of N-methyl-D-aspartate receptor (NMDAR) subunits in DH and VH. Evaluation of NR1, NR2A and NR2B mRNA levels in dorsal** (A)** and ventral **(B)** hippocampus. Data were analyzed by 2^−ΔΔCt^ using *β-actin* as normalizer. Levels of NR1, NR2A and NR2B protein levels were evaluated in total extract of dorsal **(C)** and ventral **(D)** hippocampus. Graphs represent mean ± SEM of each group. Number of animals is inside bars. **P < 0.01; *P < 0.05, two-tail Mann-Whitney test.

We found that chronic stress rises NR1 mRNA levels in DH (Figure 8A, *P* < 0.05), but not in VH (Figure 8B), compared to controls. In contrast, NR2A and NR2B mRNA levels were not affected by stress in both hippocampal areas (Figures 8A,B). We assessed whether chronic stress differentially modified PSD-95 protein levels, which is a major scaffolding protein of the postsynaptic density. We found that PSD-95 relative levels were significantly reduced both in DH (*P* < 0.05; Figure 8C) and VH (*P* < 0.05). In contrast to the rise in mRNA levels, we detected a significant reduction in protein levels of both NR1 (20%, *P* < 0.05) and NR2A (40%, *P* < 0.005) subunits in DH (Figure 8C), along with a rise of NR2B levels (Figure 8C, *P* < 0.05). The effect of stress was different in VH; only the levels of NR1 were reduced, indicating that NR2A and NR2B levels are not sensitive to stress in this hippocampal pole (Figure 8D).

**Figure 9:**
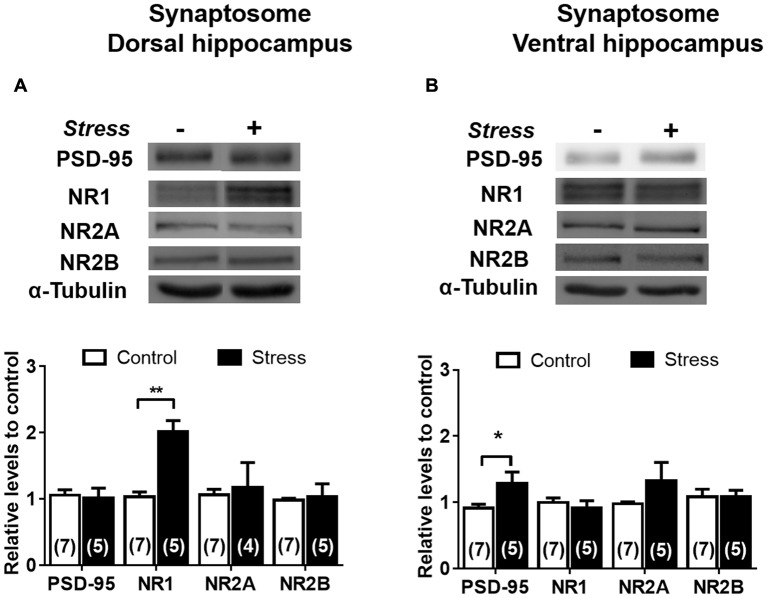
Effect of chronic stress on NMDAR subunit levels in synaptosomal fraction of DH and VH. Levels of NR1, NR2A and NR2B subunits were evaluated in synaptosomal fraction obtained from dorsal **(A)** and ventral **(B)** hippocampus. Upper panel shows a representative western blot. Graphs represent mean ± SEM of each group. Number of animals is inside bars. *P < 0.05; **P < 0.01; two-tail Mann-Whitney test.

**Figure 10:**
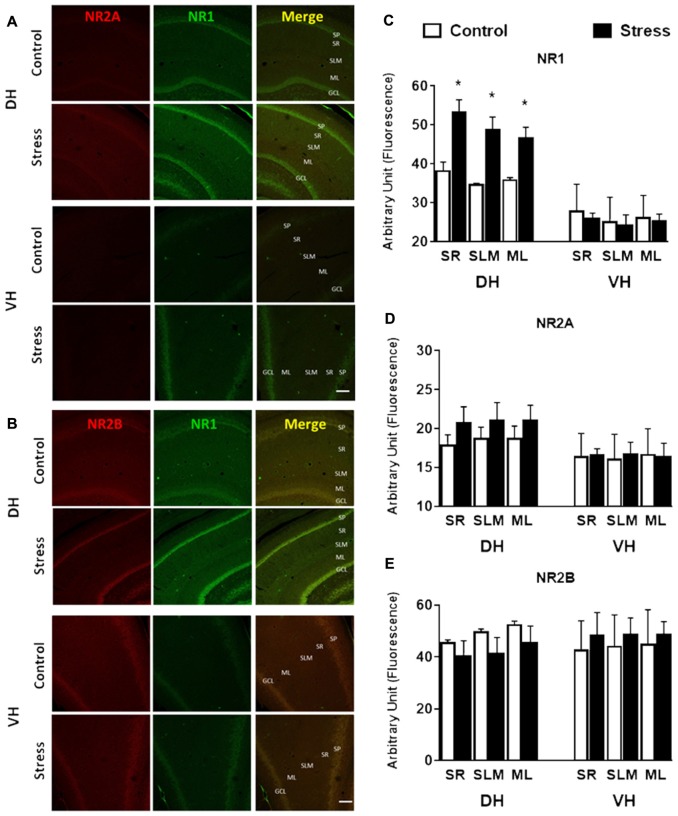
Effect of chronic stress on NMDAR subunit immunoreactivity of DH and VH. Immunoreactivity for NR1 and NR2A **(A)** and NR1 and NR2B **(B)** subunits were evaluated in DH and VH. Confocal microscopic images displaying immunoreactivity of NR2A/B (red)/NR1 (green) in DH and VH of control and chronically stressed animals. Additionally, a photograph showing the merge is shown. SP, *stratum pyramdalis*; SR, *stratum radiatum*; SLM, *stratum lacunosum moleculare*; ML, molecular layer; GCL, granular cell layer. Scale bar = 200 μm. Graphs represent the relative changes in fluorescence for NR1 **(C)**, NR2A **(D)** and NR2B **(E)**. Data represent mean ± SEM of 3–4 animals for each group. *P < 0.05; one-tail Mann-Whitney test.

In contrast to the reduction detected in whole extract, the levels of PSD-95 in DH synaptosomes were insensitive to stress (Figure 9A); nonetheless a rise (*P* < 0.05) was detected in VH synaptosomes (Figure 9B). Contrary to the reduction observed in DH whole extract, a two-fold increase in NR1 levels (*P* < 0.01) was detected in its corresponding synaptosomal fraction (Figure 9A). Furthermore, the variations in NR2A and NR2B levels detected in DH whole extracts were not correlated with similar changes in synaptosomal fraction (Figure 9A). Additionally, PSD-95 levels increased in VH synaptosomal fraction, but NR2A and NR2B levels were insensitive to chronic stress (Figure 9B). In order to visualize variations of NMDA subunits in specific areas of the hippocampus, indirect immunofluorescence was conducted. As shown in Figures 10A,B, chronic stress promoted a significant rise in the immunofluorescence of NR1 subunits in the *stratum radiatum* (SR), *stratum lacunosum moleculare* (SLM) and molecular layer (ML) of DH, but not in VH. Immunofluorescence quantifications indicate that stress promotes NR1 location in dendritic areas (Figure 10C). Furthermore, the other NMDAR subunits did not variate in SR, SLM and ML, neither in DH nor VH (Figures 10D,E).

## Discussion

In order to regulate physiological, cognitive, behavioral and emotional characteristics of the stress response, the hippocampus participates by recognizing, integrating and processing stress-related information to maintain homeostasis. Lesioning studies have revealed that DH and VH have different connectivity and hence, differ in their functions. For instance, DH has a pivotal role in cognitive functions, including spatial learning (Moser et al., 1993) and memory in rodents (Pothuizen et al., 2004), and episodic memory in humans. In contrast, VH is involved in emotional and motivated behaviors through its connection with limbic structures such as the nucleus accumbens, amygdala and hypothalamus (Moser and Moser, 1998; Kjelstrup et al., 2002).

The stress-induced disruption of DH and VH circuitries may be responsible for the ample diversity of symptoms that characterize stress-related disorders, such as depressive disorder; in which emotional alterations are commonly accompanied by cognitive dysfunctions. Many studies have shown that the intensity, duration and the chronicity of exposure to a stressor determine how the hippocampus responds to this challenge (Joels and Baram, 2009). Nonetheless, only a few studies have explored whether stress responses among DH and VH portions are similar or not. In the present study, we evaluated how chronic stress affects expression of IEG and markers of glutamatergic neurotransmission and contrasted the effect in DH and VH. We evidenced that chronic restraint triggers a rise in mRNA levels of c-Fos and Arc early genes in both DH and VH. In contrast, we detected that immunoreactivity for c-Fos is reduced in soma. Interestingly, although chronic stress did not variate the protein levels of Arc in whole extract (not shown), it probably favors its location in dendritic arbor. In relation to AMPA subunits, chronic stress did not trigger any change either in DH or VH areas. In contrast, stress triggered a reduction in PSD95 and NR1 protein levels in whole extract of DH and VH. Moreover, a reduction in NR2A/NR2B ratio was detected in DH, but with no similar variation in VH. In synaptosomal fractions we detected a rise in NR1 and PSD-95 in DH and VH, respectively. The variation in synaptosomal NR1 levels correlated with an increase in its immunoreactivity in hippocampal areas enriched in synapses.

### Chronic Stress Differentially Modifies the Magnitude of the Resting Level of IEG mRNAs in Dorsal and Ventral Hippocampus

The IEGs are classified according to their function as regulatory transcription factors, which control gene expression of other genes; and as effector IEGs, which directly impact cellular functions (Kubik et al., 2007). They are quickly expressed in response to neuronal activity, such as high-frequency stimulation that triggers LTP, and that is also observed subsequent to a behavioral experience (reviewed Kubik et al., 2007). We selected one member of each class of IEG (c-Fos and Arc) and observed an increased expression of both mRNAs in DH and VH of animals acutely stressed during 0.5 h. This response was not observed in animals acutely stressed for 2.5 h indicating that mRNAs are rapidly degraded. Additionally we did not observe a significant variation in c-Fos protein levels during of after a acute stress session. Studies have shown a relative instability of c-fos mRNA, along with a negative feedback loop by means c-Fos down–regulates its own transcription, producing low constitutive levels of c-Fos (Gius et al., 1990). Furthermore, Arc protein levels seems to be more insensitive to acute stress, although we detected a slight reduction in the immuroreactivity at the GCL of ventral DG. Thus, it is possible that acute stress in some way affect the stability of Arc.

In contrast to this pattern, in chronically stressed animals we observed a rise in the mRNAs of both IEG 24 h after the last stress session. However, the variation was more prominent in the DH, indicating that both areas display a differential responsiveness to homotypic stressor exposure. The increase in c-Fos and Arc mRNA levels, observed 24 h after the last stress exposure (daily stress during 14 days), may suggest that animals did not adapt to the repetitive restraint stress. A study shows that c-Fos mRNA response to repeated stress, measured immediately after each stress session, occurs in a region- and stressor-specific way, being the adaptation of hippocampus more gradual than cortex, hypothalamus, septum, or brainstem (Melia et al., 1994). Even though we did not address the responsiveness of hippocampus to an additional stress session, we measured c-Fos levels after the stress recovery period (24 h later), observing a new basal condition that may explain the dampened stress response reported by Melia et al. (1994).

In relation to Arc, it has been shown that repeated stress exposure produces an adaptation characterized by a reduction in Arc mRNA levels, phenomena evidenced immediately after the last 12th stress session in cingulate, infralimbic and prelimbic cortices, while piriform cortex, septum and medial and baso-lateral amygdala did not show any kind of adaptation (Ons et al., 2010). Furthermore, the literature reports variation of Arc mRNA levels in the recovery period of stress (i.e., 24 h after the last stress session). Our findings are similar to those found in a study that showed a significant increase in Arc mRNAs in hippocampus 24 h after the last session of repeatedly stress in mice (Boulle et al., 2014). In contrast to our findings, a study using chronic mild stress procedure detected a significant reduction of Arc mRNAs in frontal and cingulate cortex, and in CA1 neurons of hippocampus, but not in other hippocampal subfields (Elizalde et al., 2010). Interestingly, it has been documented that Arc mRNA is delivered and accumulated in dendrites, where it is locally translated into protein and participates in several functions, including synaptic plasticity, regulation of AMPAR internalization and structural dendritic spine remodeling (Reviewed Steward et al., 2015). In this context, the new basal condition after chronic stress that we hereby report, with increased levels of Arc mRNA, may favor more specific local changes when hippocampal functioning is required. Although the findings reported respect to stress-induced effect in levels of Arc levels are dissimilar (reviewed Li et al., 2015), we detected a rise in the immunoreactivity for Arc in DH and VH associated to dendritic layers. Thus, the effect of a higher presence of this protein in this compartment should be clarified. It would also be important to precise whether the variation observed explains some cognitive impairment observed in this chronic stress model. We concluded that repeated stress modifies the resting levels of both c-Fos and Arc mRNAs, with a different extent in DH and VH.

Preclinical studies have indicated that chronic stress exerts detrimental effects on excitatory synapses and on brain function in diverse areas related with cognitive and emotional control of reward behaviors, which resemble those observed in depressed individuals. In previous studies, others and we have described structural changes in hippocampus in response to chronic stress exposure (Magariños and McEwen, 1995; Castañeda et al., 2015; Pinto et al., 2015; García-Rojo et al., 2017). These include reduction in the number of dendritic spines in CA1 neurons of the DH (Castañeda et al., 2015; García-Rojo et al., 2017) associated with altered synaptic remodeling, which suggests altered glutamatergic circuitry. Some studies have highlighted a possible role for glutamate receptor subunits in stress-induced depressive-like behavior susceptibility (Duman, 2014).

### Chronic Stress Differentially Affects Both PSD-95 and NMDA Receptor Subunit Levels in Dorsal and Ventral Hippocampus

In our study, we have evaluated the effect of stress on the expression of PSD-95, which is almost exclusively located at the post-synaptic density of neurons (Hunt et al., 1996), and is involved in the anchoring of synaptic proteins and both AMPA and NMDARs (Sheng and Sala, 2001). In the present study, PSD-95 was significantly reduced in whole extract obtained from the DH and VH. We also evaluated protein levels at the synaptic sites using synaptosomal fractions and observed an increase of PSD-95 levels only in VH. Other studies using chronic unpredictable stress in mice have described a reduction in PSD-95 levels at the SLM, but not at SR at CA1 (Kallarackal et al., 2013); suggesting a modification of the synapses formed by entorhinal cortical axons and CA1 dendrites. Thus, variation in the levels of PSD-95 may account differences in synapse stability (Taft and Turrigiano, 2014), perhaps representing the reduction in dendritic spines in DH, in contrast to more stable synapses in VH.

NMDAR subunit levels were evaluated in whole extracts and at the synaptic sites, using synaptosomal fractions. In total extract of DH, we found that chronic stress only increases NR1 mRNA, the obligatory subunit for NMDAR, and reduces NR1 and NR2A protein levels, while NR2B rises. Similarly, to DH, we found a reduction in NR1 subunit, but without any variation in the accessory NMDA subunits. In spite of these global changes in whole extracts, we have found a dissimilar variation in synaptosomal fraction characterized by a strong increment of NR1 only in DH. Immunofluorescence experiments allow us to detect a rise in NR1 immunoreactivity at SR, SLM and ML. It is important to mention that this NMDAR subunit is normally produced in excess and is not a limiting factor in the number of NMDAR complexes being transported from the endoplasmic reticulum to the synapse (Stephenson et al., 2008). The elevated NR1 mRNA in whole extract, without the concomitant increase of total NR1 protein, may be interpreted as an increased pool of translation-repressed transcripts available for local translation. In fact, NR1 transcripts are dendritically targeted and locally translated under appropriate synaptic activity. For instance, cultured hippocampal neurons under increased neuronal activity promote the expression of NR1 and NR2A subunits at the synaptic level, an effect blocked by protein synthesis inhibitor (Swanger et al., 2013). Therefore, we propose that stress triggers activation of IEG probably by incremented neuronal activity, which may induce both synthesis and insertion of NMDAR subunits. Our results permit to envisage that chronic restraint stress locally modifies NMDAR levels and its subunit composition, an effect that can be mediated by local translation, for instance. This idea may explain the finding of the variation in NR1 immunoreactivity detected at the neuropil of DH. We found an opposite effect of chronic stress in the level of accessory subunits NR2A and NR2B in DH whole extract; variation that was not reflected in the synaptosomal fraction or in the neuropil strata of both hippocampal poles. This finding may suggest that stress has differential actions in somatic and synaptic compartments. Under the physiological point of view, the proportion of NR2A and NR2B is determinant for the electrophysiological and pharmacological properties of the channel, so these results may have a functional consequence that we have not yet explored. Furthermore, there is ample evidence that NMDAR is regulated by phosphorylation changing not only its function (Chen and Roche, 2007) but also its surface expression (Lu et al., 2015). Thus, it should be important evaluate whether stress may influence the phosphorylation of glutamate receptor subunits.

Few studies have analyzed the effect of chronic stress on NMDA subunit levels. However, these studies were conducted in whole hippocampal extract, without dissecting dorsal from ventral parts. Additionally, these studies are conflicting because one report did not find changes in NR2A and NR2B, with no evaluation of NR1 levels (Yilmaz et al., 2011), whereas another reported a significant increase in NR2A, but with no changes in neither NR2B nor NR1 levels (Pochwat et al., 2014). A recent study using proteomic approach of whole hippocampus extract obtained from male rats exposed to chronic unpredictable stress found a reduction in NR1 and a rise in NR2B levels (Ning et al., 2017); variations that are in agreement with our data obtained in DH. On the other hand, to our knowledge, only one study described a differential response of DH and VH, using chronic variable stress in rats during six consecutive weeks. The study reported a twofold rise in NR1 mRNA only in VH, although not correlated with the respective protein levels (Calabrese et al., 2012). Our data disagree with these published studies; however, the discrepancies could be explained by the use of different stress paradigms. For example, chronic unpredictable stress during six consecutive weeks used by Calabrese (Calabrese et al., 2012) vs. chronic homotypic stress performed during two weeks, paradigms that probably trigger a dissimilar physiological response.

### Chronic Stress Increases GluA1 AMPA Glutamate Receptor Subunit Only in Ventral Hippocampus

Some studies have posed that variation in hippocampal AMPAR expression may contribute to stress vulnerability in animals. For instance, using a mice model that shows stress vulnerability and depression-like symptoms, it was shown that GluA1 mRNA levels decrease and that GluA2 mRNAs levels rise in DH in comparison to resilient mice; variation accompanied by an increase in overall AMPAR binding, measured with radioligand autoradiography (Schmidt et al., 2010). This study proposes that a high GluR2-to-GluR1 subunit ratio in stress-vulnerable animals may favor the exposure of GluR2-containing AMPARs in the cell membrane (Schmidt et al., 2010). Studies have shown that the GluR2 subunit is a rate-limiting factor for the calcium influx after activation of these receptors, resulting in a desensitization of GluR2-containing AMPARs (Isaac et al., 2007). Furthermore, it was reported that chronic treatment with AMPA potentiators that slow the rate of receptor desensitization, triggers antidepressant-like effects in a chronic mild stress paradigm (Farley et al., 2010). In our model, chronic stress did not affect the levels of AMPAR subunits in whole extract and synaptosomal fraction from both hippocampal poles. Although we detected a rise in GluA1 transcript only in VH, with no variation in its protein, this variation could be biologically relevant because it may represent a pool of transcripts that are available for a stimulus-induced translation under certain conditions. In this context, it is pertinent to mention that GluA1 and GluA2 mRNAs are detected at the neuropils (Grooms et al., 2006). Thus, according to our results, we propose that chronic stress may increase GluA1 mRNAs levels in a translationally repressed pool.

### Variation in NMDA Receptor Subunits and its Relation to the Stress-Induced Altered Function in Hippocampus

It is well known that major depressive disorder is associated with an impairment of episodic memory related to hippocampal formation and is also related to anxiety (Austin et al., 2001; Drevets et al., 2008; Price and Drevets, 2010), indicating an altered function of posterior and anterior hippocampus in humans, equivalent to DH and VH in rodents. If we consider the global functional significance of our study, we must first analyze whether the functional properties of hippocampal circuits are dissimilar in dorsal and ventral parts. A recent study that carried out a morphological characterization found that ventral CA1 neurons have longer apical dendrites with fewer dendritic branches in SR, compared to dorsal pole (Malik et al., 2016). By mapping electrophysiological properties in neurons, this study also reported a gradual gradient of excitability along the dorso-ventral axis, indicating that the ventral portion is more excitable (Malik et al., 2016). This disparity in morphology and excitability in both hippocampal areas, probably will impact the behavioral and functional segregation from dorsal to ventral parts. In this context, it is important to highlight that VH is characterized by larger and less precise place field (Jung et al., 1994) and theta oscillations with less amplitude and coherence (Patel et al., 2012). Synaptic plasticity also differs along the dorso-ventral axis, where DH displays a higher threshold for LTP induction in CA1 neurons (Malik and Johnston, 2017), but controversial data has been described (Papatheodoropoulos and Kostopoulos, 2000). Moreover, how these parameters change under stress has not been systematically studied. To our knowledge, there is only one report that has compared the effect of chronic stress on dorsal and ventral plasticity; it showed that chronic unpredictable stress produced only a subtle decrease of long-term depression (LTD) maintenance in ventral CA1, without evident changes in the corresponding dorsal region (Pinto et al., 2015). In the majority of cases, only the dorsal portion was evaluated, showing a cell type-specific altered synaptic plasticity mechanism after chronic stress. Using the chronic unpredictable stress paradigm (21-days), another study showed reduced LTP in CA1 and DG (Alfarez et al., 2003). Additionally, in rats under chronic restraint stress (21 days, 6 h/day) and tested for LTP 48 h following the last stress session, LTP is suppressed in a site-specific manner (Pavlides et al., 2002). While they observed a significantly reduced LTP in the medial perforant input to the DG and commissural/associational input to the CA3, they did not observe any variation in the mossy fiber input to CA3 (Pavlides et al., 2002).

In the present study we observed that chronic stress promotes an increase in the level of synaptic NR1 and immunoreactivity in the neuropil of DH. This variation was accompanied by a negative shift in NR2A/NR2B balance in whole extract of DH. It is possible to postulate that stress influences some mechanisms related to synaptic receptor destination, favoring the insertion of NMDARs containing NR2A subunits and reducing those containing NR2B. Increased NR1 at synaptic compartment may increase the NMDAR/AMPAR ratio, influencing the excitability of DH. Moreover, the presence of a particular NMDAR subunit influences the electrophysiological properties of the channel; i.e., changing glutamate affinity and deactivation kinetics (Monyer et al., 1994; Vicini et al., 1998; Cull-Candy et al., 2001). For example, NR2A overexpression produces a compression in the range of LTD in CA1 hippocampus, specifically abolishing LTD induced in the range of 3–5 Hz, without effect in neither 1 Hz-induced LTD nor 100 Hz-induced LTP (Cui et al., 2013). On the other hand, excess of NR2B produces increased LTP, with enhanced spatial learning (Wang et al., 2009). Interestingly, although chronic stress has no effect on basal synaptic strength at Schaffer collateral synapses in the CA1 area, it impairs LTP (Alfarez et al., 2003). On the other hand, chronic stress enhances NMDAR-mediated excitatory synaptic currents in the commissural/associational input to CA3 hippocampal area, while those currents related to AMPAR remain unaltered (Kole et al., 2002). These results are also in line with ours, indicating that AMPA subunit expression is practically insensitive to chronic stress in both DH and VH. Additionally, in nucleus accumbens, increased NMDAR/AMPAR-current ratio makes synapses weaker (Thomas et al., 2001). Thus, our findings may underlie the reduced LTP reported in DH after chronic stress (Pavlides et al., 2002; Alfarez et al., 2003). Although we do not know whether subunit stoichiometry of NMDARs changed at synaptic sites, we can suggest that the reduction in PSD-95 may be indicative of the reduction in dendritic spines of CA1 subfield previously observed in our stress model (Fernández-Guasti et al., 2012; Castañeda et al., 2015; García-Rojo et al., 2017).

## Conclusions

This study provides evidence that in rats, chronic stress affects the expression of IEG and relocates Arc to dendritic compartment in DH and VH. Furthermore, chronic stress differentially alters the expression of NMDARs in VH and DH. This would indicate that restraint stress influences homeostatic synaptic plasticity related to glutamate neurotransmission, which may explain the morpho-functional alterations observed in stress-related disorders. Furthermore, dissimilar susceptibility to chronic stress in its dorsal and ventral portion opens new areas of research to understand how changes in glutamate receptor abundance may impact the output signaling from hippocampus, explaining some specific stress related symptoms. Finally, future work should prove whether these stress-induced modification are restored by antidepressant treatments.

## Author Contributions

JLF, AP, FIA and EA participated in the design of the study, analysis and interpretation of data and wrote the article, gave final approval of the version to be published; and agreed to be accountable for all aspects of the work in ensuring that questions related to the accuracy or integrity of any part of the work are appropriately investigated and resolved; AP, FIA, EA and FAO. performed the experiments, analyzed the data, participated in the drafting of article, gave final approval of the version to be published; and agreed to be accountable for all aspects of the work in ensuring that questions related to the accuracy or integrity of any part of the work are appropriately investigated and resolved; MM, GG-R, AG-P, MT-B, CSP, PSR and NAP-F participated in the analysis of data and in the drafting of the article, gave final approval of the version to be published; and agreed to be accountable for all aspects of the work.

## Conflict of Interest Statement

The authors declare that the research was conducted in the absence of any commercial or financial relationships that could be construed as a potential conflict of interest.

## Footnotes

^1^http://imagej.nih.gov/ij

## Abbreviations

AMPA: α-amino-3-hydroxy-5-methyl-4-isoxazolepropionic acid
CORT: corticosterone
CRH: corticotropin-releasing hormone
DH: dorsal hippocampus
DG: dentate gyrus
IEG: Immediate early genes
HPA: Hypothalamic-pituitary-adrenal
LAP2a: lamina-associated polypeptide
LTD: Long-term depression
LTP: Long-term potentiation
NMDA: N-methyl-D-aspartate
VH: ventral hippocampus.
